# Evolution and loss of long-fringed petals: a case study using a dated phylogeny of the snake gourds, *Trichosanthes* (Cucurbitaceae)

**DOI:** 10.1186/1471-2148-12-108

**Published:** 2012-07-03

**Authors:** Hugo J de Boer, Hanno Schaefer, Mats Thulin, Susanne S Renner

**Affiliations:** 1 Department of Systematic Biology, Uppsala University, Norbyvägen 18 D, Uppsala, SE-75236, Sweden; 2 Harvard University, Department of Organismic and Evolutionary Biology, 22 Divinity Avenue, Cambridge, MA, 02138, U.S.A; 3 Department of Systematic Biology, Uppsala University, Norbyvägen 18 D, Uppsala, SE-75236, Sweden; 4 University of Munich (LMU), Systematic Botany and Mycology, Menzinger Str. 67, Munich, 80638, Germany

## Abstract

**Background:**

The Cucurbitaceae genus *Trichosanthes* comprises 90–100 species that occur from India to Japan and southeast to Australia and Fiji. Most species have large white or pale yellow petals with conspicuously fringed margins, the fringes sometimes several cm long. Pollination is usually by hawkmoths. Previous molecular data for a small number of species suggested that a monophyletic *Trichosanthes* might include the Asian genera *Gymnopetalum* (four species, lacking long petal fringes) and *Hodgsonia* (two species with petals fringed). Here we test these groups’ relationships using a species sampling of c. 60% and 4759 nucleotides of nuclear and plastid DNA. To infer the time and direction of the geographic expansion of the *Trichosanthes* clade we employ molecular clock dating and statistical biogeographic reconstruction, and we also address the gain or loss of petal fringes.

**Results:**

*Trichosanthes* is monophyletic as long as it includes *Gymnopetalum*, which itself is polyphyletic. The closest relative of *Trichosanthes* appears to be the sponge gourds, *Luffa*, while *Hodgsonia* is more distantly related. Of six morphology-based sections in *Trichosanthes* with more than one species, three are supported by the molecular results; two new sections appear warranted. Molecular dating and biogeographic analyses suggest an Oligocene origin of *Trichosanthes* in Eurasia or East Asia, followed by diversification and spread throughout the Malesian biogeographic region and into the Australian continent.

**Conclusions:**

Long-fringed corollas evolved independently in *Hodgsonia* and *Trichosanthes*, followed by two losses in the latter coincident with shifts to other pollinators but not with long-distance dispersal events. Together with the Caribbean *Linnaeosicyos*, the Madagascan *Ampelosicyos* and the tropical African *Telfairia*, these cucurbit lineages represent an ideal system for more detailed studies of the evolution and function of petal fringes in plant-pollinator mutualisms.

## Background

**Figure 1:**
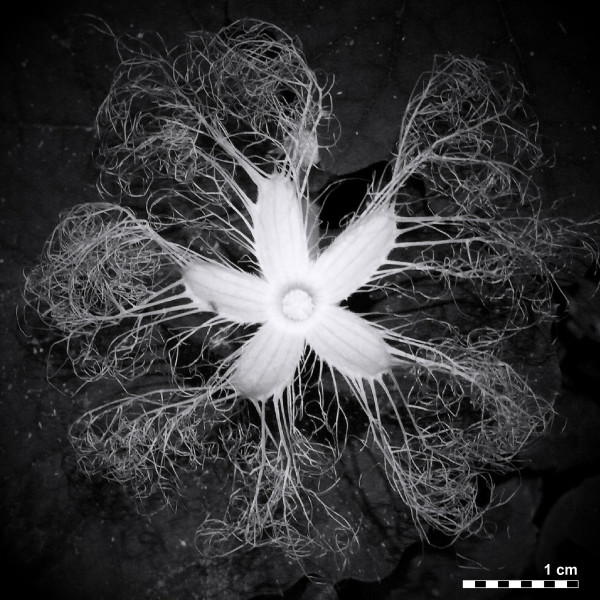
**Fully expanded flower of* Trichosanthes pilosa *Lour. showing the characteristic feather-like fringes along the petal margins.** Picture courtesy of Ken Ishikawa.

Deeply divided or fringed petal lobes are known from a range of angiosperm families, including Caryophyllaceae, Celastraceae, Cucurbitaceae, Myrtaceae, Orchidaceae, Saxifragaceae, and Tropaeolaceae [1]. While the origin and function of subdivided petals vary between groups, division of perianth edges is especially common among nocturnal hawkmoth-pollinated species (such as *Trichosanthes*[2], Figure 1), where the fringes, in combination with a light petal color, may enhance visibility and thus increase pollination success [3,4]. Experiments have shown that diurnal and nocturnal hawkmoths are attracted by floral scent but also rely on visual clues to find and recognize flowers even at extremely low light intensity [5,6]. A preference for high contrasts might help them find their nectar sources, and it seems plausible that fringed petals enhance the sharp contrast between the petal margin and a dark background [4]. 

In Cucurbitaceae, long-fringed petals are known in five genera that occur in Madagascar, tropical Africa, the Caribbean, and East and Southeast Asia [7,8]. The largest of them is *Trichosanthes* with currently 90–100 species of mainly perennial, 3 to 30 m long climbers that are usually dioecious and have medium-sized fleshy fruits. Referring to the petal fringes, Linnaeus formed the genus name from the Greek words for 'hair' (genitive τριχός) and 'flower' (Άνθoς). *Trichosanthes* has its center of diversity in Southeast Asia, but ranges from India throughout tropical and subtropical Asia east to Japan, and southeast to New Guinea, Australia, and Fiji [9]. One species, the snake gourd, *T. cucumerina* L., is a widely cultivated vegetable in tropical and subtropical regions around the globe, and another 15 species are commonly used in Asian traditional medicine [10]. While floristic treatments are available for most of its range [9,11-16], a comprehensive revision of the nearly 300 names published in *Trichosanthes* is lacking (but see [17] for a synopsis).

**Figure 2:**
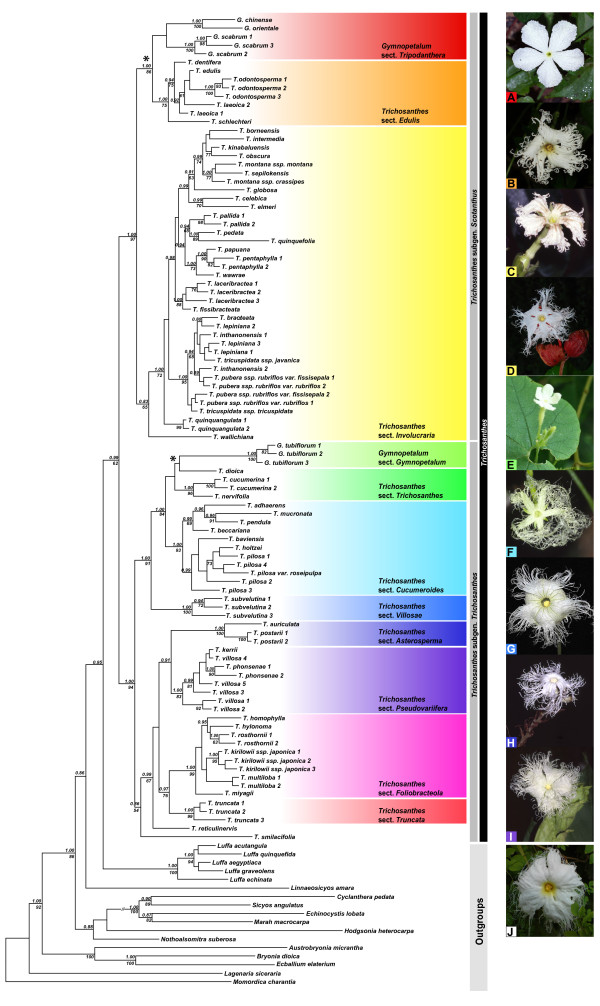
**Bayesian consensus tree with posterior probabilities (>0.80) and maximum likelihood bootstrap values (>60%) shown at the nodes.** Photos on the right illustrate the floral morphology of the different sections and belong to the following species: **A**) * Gymnopetalum chinense *; **B**) * Trichosanthes odontosperma *; **C**) * Trichosanthes montana * ssp. * crassipes *; **D**) * Trichosanthes pubera * ssp. * rubriflos *; **E**) * Gymnopetalum tubiflorum *; **F**) * Trichosanthes beccariana *; **G**) * Trichosanthes subvelutina *; **H**) * Trichosanthes postarii *; **I**) * Trichosanthes villosa *. Pictures courtesy of W. J. de Wilde and B. Duyfjes (**A**, **C**, **D**, **F**, **H**, **I**), W. E. Cooper (**B**), N. Filipowicz (**E**), H. Nicholson (**G**), and P. Brownless (**J**). Inferred losses of petal fringes are marked by an asterisk.

*Trichosanthes* belongs in the tribe Sicyoeae, a group of 12 genera and c. 270 species that is supported by morphological and molecular data [18]. Based on a limited number of *Trichosanthes* species sequenced, it appeared that the genus might be paraphyletic, with the genera *Gymnopetalum* Arn. (four species; [19]) and *Hodgsonia* Hook.f. & Thomson (two species; [9]) possibly nested inside it [20]. Both share with *Trichosanthes* the white flowers, elongated receptacle-tubes, and free filaments. *Hodgsonia* also has long-fringed petals (Figure 2J), but differs from *Trichosanthes* and *Gymnopetalum* in its much larger fruits (up to 25 cm across) and unusual seeds. The petal margins in *Gymnopetalum* are entire (Figure 2A, 2E) or in one species shortly fimbriate [9]. Geographically, *Gymnopetalum* and *Hodgsonia* largely overlap with the distribution area of *Trichosanthes* except for their absence from New Guinea and Australia, and from much of the northeastern range of *Trichosanthes* (temperate China, Taiwan, Japan) [9]. 

Based on mainly fruit and seed characters, the 43 species of *Trichosanthes* occurring in the *Flora Malesiana* region have been grouped into six sections, the typical sect. *Trichosanthes* and sections *Cucumeroides* (Gaertn.) Kitam., *Edulis* Rugayah, *Foliobracteola* C.Y.Cheng & Yueh, *Involucraria* (Ser.) Wight, and *Asterosperma* W.J.de Wilde & Duyfjes [21,22]. The mainland Asian species, *T. truncata* C.B.Clarke, is in its own section, *Truncata* C.Y.Cheng & C.H.Yueh [23]. The four species of *Gymnopetalum* have been allocated to two sections that differ in flower morphology, the typical sect. *Gymnopetalum* with just one species from southern India and Sri Lanka and sect. *Tripodanthera* (M.Roem.) Cogn. with three southeast Asian and Malesian species [24].

Here we test the monophyly and phylogenetic placement of *Trichosanthes* using a broad sampling of some 60% of its species, including the type species of each section name, plus representatives of *Gymnopetalum*, *Hodgsonia*, and other Sicyoeae as well as more distant outgroups. The well-resolved phylogeny, combined with field observations on flower shape and color, allows us to test whether petal fringes in Old World Sicyoeae evolved just once as would be the case if *Gymnopetalum* and *Hodgsonia* were nested inside it [20] or multiple times as would be implied by these genera having separate evolutionary histories. A combination of molecular-dating and ancestral area reconstruction permits reconstructing the biogeographical history of the *Trichosanthes* clade.

## Results and discussion

### Phylogenetic analyses and taxonomy

Phylogenies obtained under Bayesian or Maximum Likelihood (ML) optimization revealed no statistically supported incongruences, defined as nodes with Bayesian posterior probabilities (PP) >0.95 or ML bootstrap support >75. A Bayesian consensus tree is shown in Figure 2. It reveals that the genus *Trichosanthes* is paraphyletic because *Gymnopetalum* is embedded in it, while *Gymnopetalum* is polyphyletic because its four species do not group together. Instead, *G. tubiflorum* (Wight & Arn.) Cogn. groups with species from sections *Trichosanthes* and *Cucumeroides* (1.00 PP/84 ML support), while *G. orientale* W.J.de Wilde & Duyfjes, *G. chinense* (Lour.) Merr., and *G. scabrum* (Lour.) W.J.de Wilde & Duyfjes are sister to section *Edulis* (1.00 PP/86 ML). The *Trichosanthes*/*Gymnopetalum* clade (56 species sampled; 0.99 PP/62 ML support) is sister to *Luffa*, a genus of seven or eight species of which we included five. This sister group relationship, however, is only weakly supported (Figure 2). The genus *Hodgsonia* (two species with long-fringed flowers, one sampled here) is only distantly related to the *Trichosanthes*/*Gymnopetalum* clade.

Of the seven sections previously proposed in *Trichosanthes* (see *Background*), three are supported by the molecular results, namely sections *Asterosperma* (1.00 PP/100 ML; three species, two of them sampled here), *Cucumeroides* (1.00 PP/93 ML; seven species, five sampled), and *Edulis* (1.00 PP/75 ML; nine species, five sampled). Three other sections with more than one species (*Involucraria*, *Foliobracteola*, *Trichosanthes*) are not monophyletic in their current circumscriptions. To achieve a more natural classification, a revised infrageneric classification has been proposed including two new sections [17].

### The biogeographic history of the Trichosanthes clade

**Figure 3:**
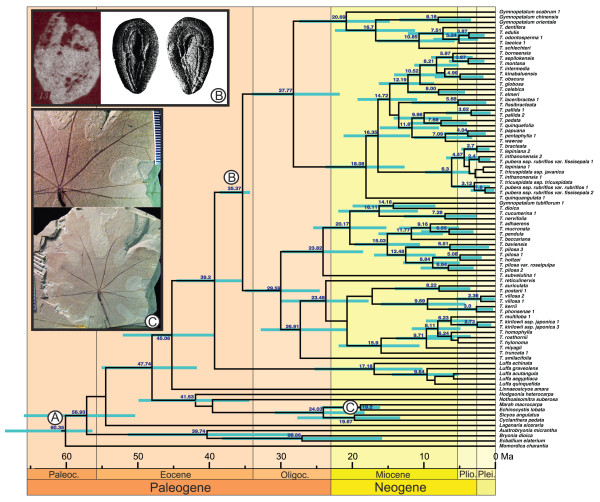
**Chronogram for* Trichosanthes *and outgroups obtained from the same sequence data as used for Figure**1**, but modeled under a relaxed molecular clock.** Node heights represent mean ages and bars the 95% highest posterior density intervals for nodes that have a posterior probability of ≥ 0.95. Fossil constraints used were: (**A**) Cucurbitaceae seeds from the London Clay (see * Material and Methods *), (**B**) * Trichosanthes * seeds from Eocene sediments in Bulgaria [25] and Oligocene sediments in West Siberia [26], and (**C**) Miocene leaves assigned to *Marah*. Inset B shows the Bulgarian seeds ([25], Figure thirteen) to the left and Middle Pliocene seeds from Poland ([29], Figures sixteen to seventeen) to the right: Inset C shows the * Marah * leaf (photos provided by M. Guilliams and D.M. Erwin, University of California, Berkeley).

**Figure 4:**
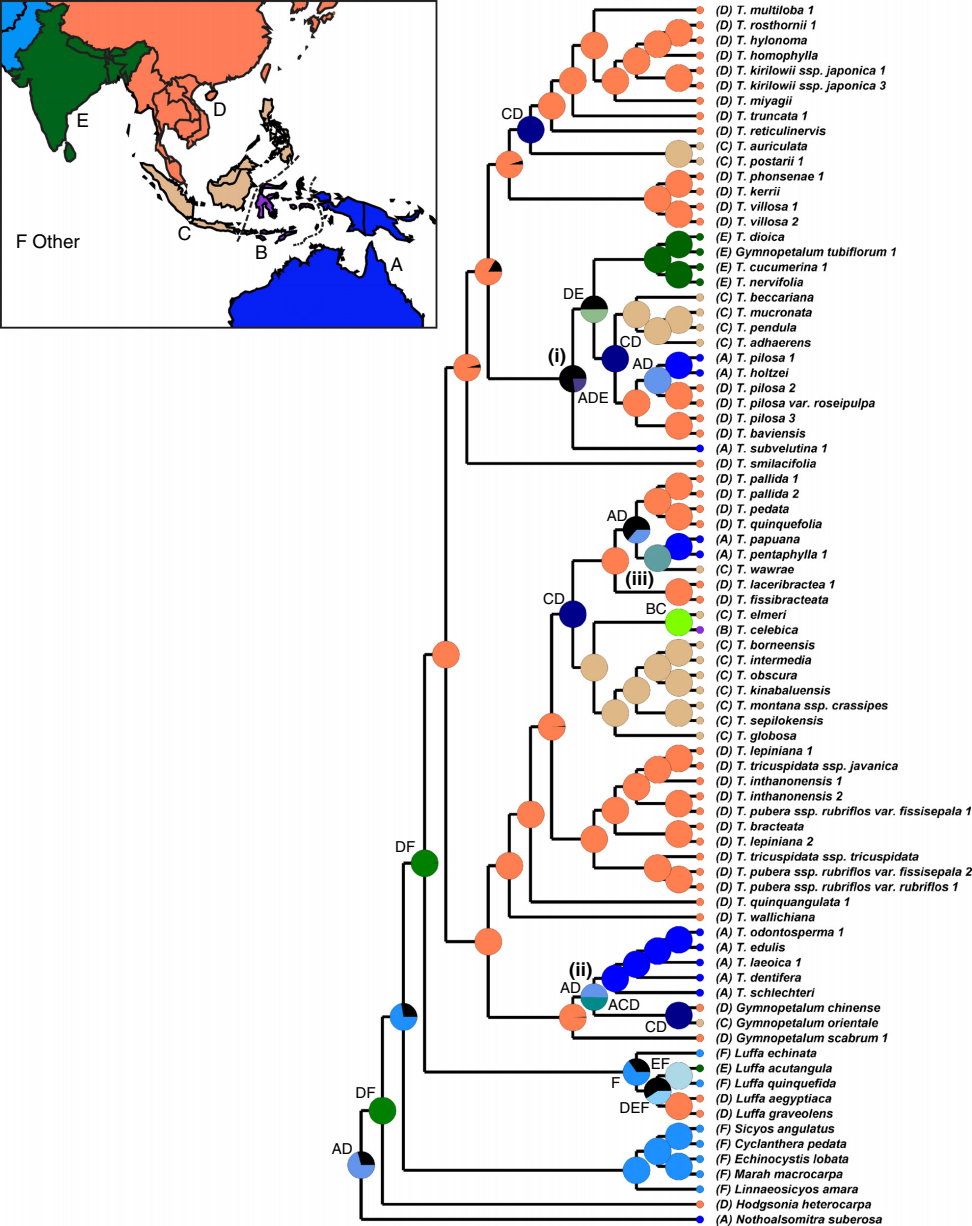
**Ancestral range reconstruction for* Trichosanthes *and outgroups inferred on 8000 output trees resulting from the Bayesian dating analysis and distribution ranges for all species.** Letters in the legend correspond to the colored distribution ranges in the map (inset), and letters adjacent to taxon names correspond to the geographic origin of the sampled plant. Wallace’s Line is shown as a broken line between Borneo and Sulawesi, Lydekker’s Line is shown as a broken line between New Guinea and the Moluccas. The three numbered clades and inferred transoceanic disjunctions are discussed in the text.

Based on a fossil-calibrated Bayesian relaxed molecular clock model, *Trichosanthes* originated during the Oligocene (Figure 3), an estimate influenced by our prior constraint of the crown node of the *Trichosanthes*/*Gymnopetalum* clade to 34 Ma. This constraint is based on *Trichosanthes*-like seeds from the Upper Eocene of Bulgaria [25] dating to c. 34 Ma and seeds from the Oligocene of West Siberia [26] dating to c. 23.8 Ma [27]. Seeds assigned to *Trichosanthes* have also been reported from Miocene and Pliocene sites in France, Germany, Italy, and Poland [28-30], and Pliocene *Trichosanthes*-like leaves are known from France [31]. The biogeographic analysis (Figure 4) inferred an East Asian origin of the genus (region C in Figure 4), but this inference is based only on the living species, while the just-discussed fossils indicate a more northern (Eurasian) range of *Trichosanthes* before the global climate cooling at the end of the Oligocene. Many other extinct elements of the European Oligocene, Miocene, and Pliocene floras, such as *Taxodium*, *Craigia*, *Fagus kraeuselii*, *Ilex*, and tropical Araceae, such as *Caladiosoma*, also have nearest living relatives in tropical Southeast Asia [31,32]. 

Collision between the Eurasian and Australian tectonic plates started in the Late Oligocene, about 25 Ma ago, and the Sahul Shelf (carrying New Guinea) and Sunda Shelf (Sumatra, Java, and Borneo) reached their present proximity only by the Late Miocene, some 10 Ma [33,34]. Mid-Miocene pollen records indicate a warm, moist climate and rainforest expansion on these newly forming islands [35], allowing groups adapted to humid forest conditions, such as the liana clade *Trichosanthes*, to spread and diversify. Such plant groups would have benefited from land bridges that during times of sea level changes repeatedly connected New Guinea and Australia on the one hand, and Indochina, Sumatra, Java, and Borneo on the other. The lowest sea levels, during the last glacial maximum (LGM), were approximately 120 m below those of today, resulting in the complete exposure of the Sunda Shelf; even sea level reduction by just 40 m already connected Indochina, Sumatra, Java, and Borneo [35,36]. No land bridges, however, ever connected the islands on the Sunda Shelf with those in “Wallacea,” that is, Sulawesi, the Moluccas, and the Lesser Sunda Islands, or the latter with New Guinea and Australia on the Sahul Shelf. In zoogeography, these two boundaries are known as Wallace’s Line and Lydekker’s line, but their significance as floristic boundaries is doubtful [37,38].

The most striking transoceanic disjunctions in *Trichosanthes* are numbered in Figure 4. They are (i) the disjunction between the Australian species *T. subvelutina* F.Muell. ex Cogn. and its sister clade on the Asian mainland and areas of the Sunda Shelf, dated to 23.8 (29.4-18.4) Ma; (ii) the disjunction between *T. edulis* Rugayah*, T. dentifera* Rugayah*, T. laeoica* C.Y.Cheng & L.Q.Huang*, T. schlechteri* Harms from New Guinea, and *T. odontosperma* W.E.Cooper & A.J.Ford from Australia on the one hand, and *Gymnopetalum chinense*, widespread in Asia as far East as Flores, and *G. orientale* in Sulawesi, the Lesser Sunda Islands, and the Moluccas on the other (this is dated to 16.7 (22.1-11.2) Ma, but the position of *G. scabrum* relative to *G. chinense* and *G. orientale* remains unclear; compare Figures 2, 3, and 4); and (iii) the disjunction between *T. wawrae* Cogn. from Thailand, peninsular Malaysia, Sumatra, and Borneo, and its sister clade *T. papuana* F.M.Bailey/*T. pentaphylla* F. Muell. ex Benth. from New Guinea and Australia, which dates to 7.1 (11.2-3.3) Ma.

*Trichosanthes* range expansion between New Guinea and Australia occurred during the Pliocene/Pleistocene, when these two regions were repeatedly connected due to the above-mentioned sea level changes [36]. Thus, the estimated divergence time of the Australian species *T. odontosperma* (a member of clade ii in Figure 4) from its New Guinean sister species, *T. edulis*, is 3.9 (6.4-1.6) Ma, while that of the sister species pair *T. papuana* from the Aru Islands and New Guinea, and *T. pentaphylla* from Australia (clade iii in Figure 4) is 4.0 (7.1-1.4) Ma; considering their error ranges, these ages fall in the Pliocene/Pleistocene.

The geographic history of *T. pilosa* Lour. (including the synonyms *T. baviensis* Gagnep. and *T. holtzei* F.Muell. [16]), a widespread species here represented by seven samples from Queensland (Australia), Thailand, Vietnam, and Japan, cannot be inferred because the within-species relationships lack statistical support (Figure 2). Inferring the origin of the snake gourd, *T. cucumerina*, a vegetable cultivated in tropical and subtropical regions around the globe (represented by a single sample from Sri Lanka) also would require population-level sampling. Both species have fleshy red fruits and small seeds, probably dispersed by birds.

### Evolution and loss of petal fringes

The phylogeny obtained here implies that long-fringed corollas evolved independently in the Asian genera *Hodgsonia* and *Trichosanthes* and were lost in three of the four species formerly placed in the genus *Gymnopetalum* (petals still bear c. 5 mm-long fringes in *G. orientale*). The two inferred losses (marked with an asterisk in Figure 2) coincide with shifts from nocturnal to diurnal flowering times (HS personal observation of *G. scabrum* and *G. chinense* in Cambodia, Jan. 2010, and China, Sept. 2005; N. Filipowicz, Medical University of Gdansk, personal observation of *G. tubiflorum* in India, Nov. 2010), and it therefore seems likely that there is a shift from predominantly nocturnal sphingid pollinators to diurnal bee or butterfly pollinators. The loss of fringes does not coincide with long-distance dispersal events to insular habitats (where hawkmoths might be absent), and the trigger for the pollinator shifts so far is unknown.

The adaptive function of the corolla fringes in pollinator attraction requires experimental study. An innate preference for radial patterns [39] and high contrasts might help hawkmoths find their nectar sources [5,6], and one possible explanation for the evolution of fringed petals is that they help create such a radial pattern and sharper contrasts between the petals and a dark background [4]. In a diurnal, hawkmoth-pollinated *Viola* species, more complex corolla outlines correlate with higher fruit set [40] but it remains to be tested if this is also the case in the nocturnal *Trichosanthes*-hawkmoth system. Another untested possibility is that the fringes with their highly increased surface area and exposed position might be involved in scent production (B. Schlumpberger, Herrenhaeuser Gardens, Hannover, pers. comm., Feb. 2012) or produce a waving motion, which has been shown to increase pollinator attraction in other systems [41]. Anatomical studies of the petal tissue of *Trichosanthes*, wind tunnel experiments with naive hawkmoths, and detailed field observations are required to test these possibilities.

## Conclusions

Molecular evidence supports the inclusion of *Gymnopetalum* into a then monophyletic *Trichosanthes*[17]*.* Our molecular phylogenies reveal that long-fringed petals evolved independently in *Hodgsonia* and *Trichosanthes/Gymnopetalum*, followed by two losses of corolla fringes in the latter clade, most likely associated with pollinator shifts. Molecular dating and a biogeographic analysis indicate an Oligocene initial diversification of *Trichosanthes* in mainland Asia. The lineage then diversified and spread in Malaysia (the Malesian biogeographic region) during the late Miocene and Pliocene, reaching the Australian continent several times.

## Methods

### Morphology

Herbarium specimens from A, BRI, CNS, E, GH, K, KUN, KYO, L, LE, M, MO, P, S, UC, UPS and US were obtained on loan or studied during herbarium visits. Determination of herbarium material was verified using identification keys [9,11-16,19,42]. All species in *Trichosanthes* have corolla fringes, and these are absent in three of the four *Gymnopetalum* species, except *G. orientale*, which can have short-fimbriate petal margins (fringes up to 5 mm length).

### Sampling, DNA extraction and amplification

We included six DNA regions, namely the nuclear ribosomal ITS region (ITS1-5.8S-ITS2), the chloroplast genes *rbcL* and *matK*, the *trnL* and *trnLtrnF* intron and spacer, and *rpl20-rps12* spacer. Data for *rbcL* and the *trnL* region were taken from previous studies [7,18,20,43,44]. Only plant samples for which two or more markers were successfully sequenced were included in the analyses, and the combined dataset included one of the two species of *Hodgsonia,* all four of *Gymnopetalum*, and 52 of *Trichosanthes*, representing approximately 60% of the accepted species in the latter genus. Type species of all sections were included: *Gymnopetalum tubiflorum* (Wight & Arn.) Cogn. (*G.* sect. *Gymnopetalum*), *Gymnopetalum chinense* (Lour.) Merr. (*G.* sect. *Tripodanthera*), *Trichosanthes postarii* W.J.de Wilde & Duyfjes (*T.* sect. *Asterosperma*), *Trichosanthes pilosa* Lour. (*T.* sect. *Cucumeroides*), *Trichosanthes edulis* Rugayah (*T.* sect. *Edulis*), *Trichosanthes kirilowii* Maxim. (*T.* sect. *Foliobracteola*), *Trichosanthes wallichiana* (Ser.) Wight (*T.* sect. *Involucraria*), *Trichosanthes villosa* Blume (*T.* sect. *Pseudovariifera*), *Trichosanthes cucumerina* L. (*T.* sect. *Trichosanthes*), *Trichosanthes truncata* C.B.Clarke (*T.* sect. *Truncata*), *Trichosanthes subvelutina* F.Muell. ex Cogn. (*T.* sect. *Villosae*). Species names and their authors, specimen voucher information, and GenBank accession numbers for all sequenced markers (including 262 new sequences) are summarized in Table 1. 

**Table 1:** Voucher information and GenBank accession numbers

**Species**	**No.**	**Voucher (Herbarium)**	**Origin of the sequenced material**	**ITS**	***rpl*20-*rps*12 IS**	***mat*K**	***rbc*L**	***trn*L-*trn*F IS**	***trn*L intron**
*Austrobryonia micrantha* (F.Muell.) I.Telford		*I. R. Telford* 8173 (CANB)	Australia, New South Wales	EF487546	EF487567	EF487559	EF487552	EF487575	EF487575
*Bryonia dioica* Jacq.		(1) *S. Renner* 2187 (M)	(1) Switzerland, cult. BG Zürich	(2) EU102709	(1) DQ648157	(1) DQ536641	(1) DQ536791	(1) DQ536791	(1) DQ536791
		(2) *A. Faure* 66/76 (M)	(2) Algeria, Lamoriciere						
*Cyclanthera pedata* (L.) Schrad.		*S. Renner* et al. 2767 (M)	Germany, cult. BG Mainz	HE661293	DQ648172	DQ536667	DQ535749	DQ536767	DQ536767
*Ecballium elaterium* (L.)A.Rich. ssp. *elaterium*		(1) *M. Chase* 922 (K)	(1) UK, cult. RBG-K	(2) EU102746	(1) AY968541	(1) AY973019	(1) AY973023	(1) AY973006	(1) AY973006
		(2) *S. Renner* et al. 2768 (M)	(2) Germany, cult. BG Mainz						
*Echinocystis lobata* (Michx.) Torr. & A.Gray		*S. Renner* et al. 2829 (M)	Germany, cult. BG Mainz	-	DQ648174	DQ536673	DQ535809	DQ536814	DQ536814
*Gymnopetalum chinense* (Lour.) Merr.		*H. Schaefer* 2005/661 (M)	China, Guangdong	HE661294	EU155612	EU155606	EU155601	EU155621	EU155630
*Gymnopetalum orientale* W.J. de Wilde & Duyfjes		*M. van Balgooy* 7553 (L)	Indonesia, Bali	HE661301	HE661468	HE661397	-	-	-
*Gymnopetalum scabrum* (Lour.) W.J. de Wilde & Duyfjes	1	*W. de Wilde & B. Duyfjes* 22269 (L)	Thailand, Central	HE661295	DQ536556	DQ536683	DQ535754	DQ536824	DQ536824
*Gymnopetalum scabrum* (Lour.) W.J. de Wilde & Duyfjes	2	*J. Maxwell* 16-11-2002 (CMU)	Thailand	HE661296	HE661469	HE661398	-	-	-
*Gymnopetalum scabrum* (Lour.) W.J. de Wilde & Duyfjes	3	*C.H. Wong, J. Helm & J. Schultze-Motel* 2071 (LE)	China, Hainan	HE661297	HE661470	HE661399	-	-	-
*Gymnopetalum tubiflorum* (Wight & Arn.) Cogn.	1	*N. Filipowicz & Z. Van Herwijnen* NF25a (M)	India, Kerala	HE661298	HE661471	HE661400	-	-	-
*Gymnopetalum tubiflorum* (Wight & Arn.) Cogn.	2	*A. Alston* 1670 (UC)	Sri Lanka, Veragantota	HE661299	HE661472	HE661401	-	-	-
*Gymnopetalum tubiflorum* (Wight & Arn.) Cogn.	3	*G.H.K. Thwaites* CP1625 (K)	Sri Lanka	HE661300	HE661473	HE661402	-	-	-
*Hodgsonia heteroclita* Hook.f. & Thomson		(1) P. *Phonsena* 4705 (L)	(1) Thailand, Nan	(1) HE661302	(1) HE661474	(1) HE661403	-	(2) EU155631	-
		(2) *L. Loeffler* s.n. (M)	(2) Bangladesh						
*Lagenaria siceraria* (Molina) Standl.		*M. Merello* 1331 (MO)	Ghana	HE661303	HE661475	HE661404	AY935747	AY935788	AY968570
*Linnaeosicyos amara* (L.) H.Schaef. & Kocyan		*M. Mejia, J. Pimentel & R. Garcia* 1877 (NY)	Dominican Republic	HE661304	DQ536602	DQ536741	DQ535774	DQ536873	DQ536873
*Luffa acutangula* (L.) Roxb.		(1) *S. Renner* et al. 2757 (M), seeds from D. S. Decker-Walters & A. Wagner TCN 1130 (FTG)	(1) Germany, cult. BG Munich, seeds from India, Ahmadnagar, Maharasthra	(1) HE661305	(1) HE661476	(2) DQ536695	(2) DQ535826	(2) DQ536835	(2) DQ536835
		(2) *L.X. Zhou* s.n., no voucher	(2) China, cult. BG Guangzhou						
*Luffa aegyptiaca* Mill. (incl. *L. cylindrica* L.)		*D.Z. Zhang* 15 April 2003, no voucher	China, cult. BG Guangzhou	HE661306	HE661477	HE661405	DQ535827	DQ536836	DQ536836
*Luffa echinata* Roxb.		*G. Schweinfurth* 555 (M)	Egypt	HE661307	HE661478	HE661406	-	EU436357	EU436357
*Luffa graveolens* Roxb.		*S. Renner & A. Kocyan* 2758 (M), seeds from D. Decker-Walters 1543 (FTG 121855)	Germany, cult. BG Munich, seeds from India, USDA PI540921	HE661308	EU436334	EU436409	EU436385	EU436358	EU436358
*Luffa quinquefida* (Hook. & Arn.) Seemann		(1) *R. Berhaut* 7308 (M)	(1) Senegal	(2) HQ201986	(1) EU436335	(2) DQ536697	-	(1) EU436359	-
		(2) *S. Renner & A. Kocyan* 2754 (M), seeds from D. S. Decker-Walters TCN 1440 (FTG 118010)	(2) Germany, cult. BG Munich, seeds originally from Louisiana, USA						
*Marah macrocarpa* (Greene) Greene		(1) *M. Olson* s.n. (MO)	(1) USA, Sonoran Desert	(2) AF11906-7	(1) DQ536566	(2) AY968453	(2) AY968524	(1) AY968387	(1) AY968571
		(2) *D. Arisa & S. Swensen* 1009 (RSA)	(2) USA, Sonoran Desert						
*Momordica charantia* L.		*S. Renner* et al. 2775 (M)	Germany, cult. BG Munich	HE661309	DQ491013	DQ491019	DQ535760	DQ501269	DQ501269
*Nothoalsomitra suberosa* (F.M.Bailey) I.Telford		*I. Telford* 12487 (NE)	Australia, SE Queensland	HE661310	DQ536575	DQ536709	DQ535762	DQ536844	DQ536844
*Sicyos angulatus* L.		*M. Chase* 979 (K)	North America	HE661311	DQ648189	DQ536732	DQ535847	DQ536777	DQ536777
*Trichosanthes adhaerens* W.J. de Wilde & Duyfjes		*S. Lim, J. J. Postar & G. Markus* SAN 143273 (L)	Malaysia, Borneo, Sabah	HE661312	HE661479	-	-	-	-
*Trichosanthes auriculata* Rugayah		*A. Kalat, I. Abdullah, & J. Clayton* BRUN 17016 (L)	Borneo, Brunei	HE661313	HE661480	HE661407	-	-	-
*Trichosanthes baviensis* Gagnep.		*N.M. Cuong* 1248 (P)	Vietnam	HE661314	HE661481	-	-	-	-
*Trichosanthes beccariana* Cogn. ssp. *beccariana*		*W. de Wilde* et al. SAN 142229 (L)	Malaysia, Borneo, Sabah	HE661315	HE661482	HE661408	-	-	-
*Trichosanthes borneensis* Cogn.		*C. Argent* et al. 93127 (E)	Indonesia, Borneo, Kalimantan Timur	HE661316	HE661483	-	-	-	-
*Trichosanthes bracteata* (Lam.) Voigt		*T. Haegele* 20 (M)	India, Kochin	HE661317	HE661484	EU155608	EU155602	EU155622	EU155632
*Trichosanthes celebica* Cogn.		*W. de Wilde & B. Duyfjes* 21903 (L)	Indonesia, Sulawesi	HE661318	HE661485	HE661409	-	-	-
*Trichosanthes cucumerina* L.	1	*H. Schaefer* 2007/327 (M)	Germany, cult. BG Munich	HE661319	EU155614	EU155609	EU155603	EU155623	EU155633
*Trichosanthes cucumerina* L.	2	*N. Lundqvist* 11380 (UPS)	Sri Lanka	HE661320	HE661486	HE661410	-	-	-
*Trichosanthes dentifera* Rugayah		*J.H.L. Waterhouse* 445-B (L)	Papua New Guinea, Bougainville Is.	HE661321	HE661487	-	-	-	-
*Trichosanthes dioica* Roxb.		*O. Polunin, W. Sykes & J. Williams* 5925 (E)	Nepal	HE661322	HE661488	HE661411	-	-	-
*Trichosanthes edulis* Rugayah		*W. Avé* 4076 (L)	Indonesia, Irian Jaya	HE661323	HE661489	HE661412	-	-	-
*Trichosanthes elmeri* Merr.		*E.F.J. Campbell* 43 (E)	Malaysia, Borneo, Sabah	HE661324	HE661490	-	-	-	-
*Trichosanthes globosa* Blume		*W. de Wilde* et al. SAN 144003 (L)	Malaysia, Borneo, Sabah	HE661325	HE661491	HE661413	-	-	-
*Trichosanthes holtzei* F.Muell.		*B. Gray* 7482 (CNS)	Australia, N Queensland	HE661326	HE661492	HE661414	-	-	-
*Trichosanthes homophylla* Hayata		*Y.-C. Kao* 499 (GH)	Taiwan	HE661327	HE661493	HE661415	-	-	-
*Trichosanthes hylonoma* Hand.-Mazz.		*Wuling Mt Exp* 1646 (KUN)	China	HE661328	HE661494	HE661416	-	-	-
*Trichosanthes intermedia* W.J. de Wilde & Duyfjes		*V. Julaihi* et al. S 76602 (L)	Malaysia, Borneo, Sarawak	HE661329	HE661495	-	-	-	-
*Trichosanthes inthanonensis* Duyfjes & Pruesapan	1	*P. Phonsena, W. de Wilde & B. Duyfjes* 3930 (L)	Thailand, Chiang Mai	HE661330	HE661496	HE661417	-	-	-
*Trichosanthes inthanonensis* Duyfjes & Pruesapan	2	*K. Pruesapan* et al. 67 (L)	Thailand, Kanchanaburi	HE661331	HE661497	HE661418	-	-	-
*Trichosanthes kerrii* Craib		*P. Phonsena, W. de Wilde & B. Duyfjes* 3959 (L)	Thailand, Nan	HE661333	HE661498	-	-	-	-
*Trichosanthes kinabaluensis* Rugayah		*J. Postar* et al. SAN 144260 (L)	Malaysia, Borneo, Sabah	HE661334	EU155615	HE661419	-	EU155624	EU155634
*Trichosanthes kirilowii* Maxim. var. *japonica* (Miq.) Kitam.	3	*H. Takahashi* 20711 (GIFU)	Japan	HE661335	DQ536603	DQ536742	DQ535855	DQ536874	DQ536874
*Trichosanthes kirilowii* Maxim. var. *japonica* (Miq.) Kitam.	1	*K. Kondo* 05090401e (KYO)	Japan	HE661332	HE661499	HE661420	-	-	-
*Trichosanthes kirilowii* Maxim. var. *japonica* (Miq.) Kitam.	2	*K. Deguchi, K. Uchida, K. Shiino & H. Hideshima* s.n. (KYO)	Japan	-	HE661500	HE661421	-	-	-
*Trichosanthes laceribractea* Hayata	1	*S. Fujii* 9623 (KYO)	Japan	HE661336	HE661501	HE661422	-	-	-
*Trichosanthes laceribractea* Hayata	2	*S. Fujii* 9978 (KYO)	Japan	HE661337	HE661502	HE661423	-	-	-
*Trichosanthes laceribractea* Hayata	3	*Liang Deng* 7090 (KUN)	China	HE661338	HE661503	-	-	-	-
*Trichosanthes laeoica* C.Y.Cheng & L.Q.Huang	1	*M. Coode* et al. NGF 32585 (E)	Papua New Guinea, Eastern Highlands	HE661339	HE661504	-	-	-	-
*Trichosanthes laeoica* C.Y.Cheng & L.Q.Huang	2	*P. Katik* LAE 77807a (BRI)	Papua New Guinea	HE661340	HE661505	-	-	-	-
*Trichosanthes lepiniana* (Naud.) Cogn.	1	*J.D.A. Stainton* 8522 (E)	Nepal	HE661341	HE661506	HE661424	-	-	-
*Trichosanthes lepiniana* (Naud.) Cogn.	2	*Shanzu Wen* 85 (KUN)	China	HE661342	HE661507	HE661425	-	-	-
*Trichosanthes lepiniana* (Naud.) Cogn.	3	*H. de Boer* HB49, coll. 1865 (P)	France, cult BG Paris	HE661343	HE661508	-	-	-	-
*Trichosanthes miyagii* Hayata		*T. Yamazaki* 310 (KYO)	Japan	HE661344	HE661509	HE661426	-	-	-
*Trichosanthes montana* Rugayah ssp. c*rassipes* W.J. de Wilde & Duyfjes		*J. Postar* et al. SAN 144259 (L)	Malaysia, Borneo, Sabah	HE661346	EU155616	HE661427	-	EU155625	EU155635
*Trichosanthes montana* Rugayah ssp. *montana*		*W. de Wilde* et al. 22279 (L)	Indonesia, Java	HE661345	HE661510	-	-	-	-
*Trichosanthes mucronata* Rugayah		*W. de Wilde & B. Duyfjes* SAN 139459 (L)	Malaysia, Borneo, Sabah	HE661347	HE661511	HE661428	-	-	-
*Trichosanthes multiloba* Miq.	1	*S. Tsugaru, G. Murata & T. Sawada* s.n. (KYO)	Japan	HE661348	HE661512	HE661429	-	-	-
*Trichosanthes multiloba* Miq.	2	*S. Fujii* 9957 (KYO)	Japan	HE661349	HE661513	HE661430	-	-	-
*Trichosanthes nervifolia* L.		*B. Jonsell* 3828 (UPS)	Sri Lanka	HE661350	HE661514	HE661431	-	-	-
*Trichosanthes obscura* Rugayah		*K.M. Wang* 1581 (L)	Borneo, Brunei	HE661351	HE661515	-	-	-	-
*Trichosanthes odontosperma* W.E.Cooper & A.J.Ford	1	*H. Schaefer* 2007/09 (M)	Australia, Queensland	HE661352	EU037013	HE661432	-	EU037011	EU037010
*Trichosanthes odontosperma* W.E.Cooper & A.J.Ford	2	*B. Gray* 9147 (UPS)	Australia, Queensland	HE661353	HE661516	HE661433	-	-	-
*Trichosanthes odontosperma* W.E.Cooper & A.J.Ford	3	*I. Telford* 11285 (CNS)	Australia, Queensland	HE661354	HE661517	HE661434	-	-	-
*Trichosanthes pallida* Duyfjes & Pruesapan	1	*P. Phonsena, W. de Wilde & B. Duyfjes* 4658 (L)	Thailand, Phetchaburi	HE661355	HE661518	HE661435	-	-	-
*Trichosanthes pallida* Duyfjes & Pruesapan	2	*P. Phonsena, W. de Wilde & B. Duyfjes* 3981 (L)	Thailand, Phetchaburi	HE661356	HE661519	HE661436	-	-	-
*Trichosanthes papuana* F.M.Bailey		*W. Takeuchi & D. Ama* 17069 (L)	Papua New Guinea	HE661357	HE661520	HE661437	-	-	-
*Trichosanthes pedata* Merr. & Chun		*Jiangiang Li* 239 (KUN)	China	HE661358	HE661521	HE661438	-	-	-
*Trichosanthes pendula* Rugayah		*J. Postar* et al. 144100 (L)	Malaysia, Borneo, Sabah	HE661359	EU155617	HE661439	-	EU155626	EU155636
*Trichosanthes pentaphylla* F.Muell. ex Benth.	1	*W. Cooper* 2094 (CNS)	Australia, Queensland	HE661360	HE661522	HE661440	-	-	-
*Trichosanthes pentaphylla* F.Muell. ex Benth.	2	*W. Cooper* 2061 (CNS)	Australia, Queensland	HE661361	HE661523	HE661441	-	-	-
*Trichosanthes phonsenae* Duyfjes & Pruesapan	1	*P. Phonsena, W. de Wilde & B. Duyfjes* 4419 (L)	Thailand, Phetchaburi	HE661362	HE661524	HE661442	-	-	-
*Trichosanthes phonsenae* Duyfjes & Pruesapan	2	*P. Phonsena, W. de Wilde & B. Duyfjes* 3980 (L)	Thailand, Phetchaburi	HE661363	HE661525	HE661443	-	-	-
*Trichosanthes pilosa* Lour.	1	*H. Schaefer* 2007/17 (M)	Australia, Queensland	HE661364	EU155620	EU155611	-	EU155629	EU155639
*Trichosanthes pilosa* Lour.	2	*P. Phonsena, W. de Wilde & B. Duyfjes* 3913 (L)	Thailand, Chiang Mai	HE661365	HE661526	HE661444	-	-	-
*Trichosanthes pilosa* Lour.	3	*H. Takahashi* 20755 (GIFU)	Japan	-	DQ536604	DQ536743	DQ535856	DQ536875	DQ536875
*Trichosanthes pilosa* Lour.	4	*H. Schaefer* 2007/09 (M)	Australia, Queensland	HE661366	HE661528	HE661445	-	-	-
*Trichosanthes pilosa* var. *roseipulpa* W.J. de Wilde & Duyfjes		*P. Phonsena, W. de Wilde & B. Duyfjes* 4694 (L, holotype)	Thailand, Nan	HE661367	HE661529	HE661446	-	-	-
*Trichosanthes postarii* W.J. de Wilde & Duyfjes	1	*J. Postar* et al. SAN 144066 (L, isotype)	Malaysia, Borneo, Sabah	HE661368	EU155618	HE661447	-	EU155627	EU155637
*Trichosanthes postarii* W.J. de Wilde & Duyfjes	2	*J. Postar* et al. SAN 144098 (L)	Malaysia, Borneo, Sabah	HE661369	HE661530	HE661448	-	-	-
*Trichosanthes pubera* Blume ssp. *rubriflos* (Cayla) Duyfjes & Pruesapan var. *fissisepala* Duyfjes & Pruesapan	1	*P. Phonsena, W. de Wilde & B. Duyfjes* 4451 (L)	Thailand, Chiang Mai	HE661370	HE661531	HE661449	-	-	-
*Trichosanthes pubera* Blume ssp. *rubriflos* (Cayla) Duyfjes & Pruesapan var. *fissisepala* Duyfjes & Pruesapan	2	*K. Pruesapan* et al. 56 (L)	Thailand, Kanchanaburi	HE661371	HE661532	HE661450	-	-	-
*Trichosanthes pubera* Blume ssp. *rubriflos* (Cayla) Duyfjes & Pruesapan var. *rubriflos*	1	*R. Zhang* 1 (M)	China, cult. South China BG, Guangzhou	HE661372	DQ536560	DQ536688	DQ535819	DQ536828	-
*Trichosanthes pubera* Blume ssp. *rubriflos* (Cayla) Duyfjes & Pruesapan var. *rubriflos*	2	*P. Phonsena, W. de Wilde & B. Duyfjes* 3907 (L)	Thailand, Saraburi	HE661373	HE661533	HE661451	-	-	-
*Trichosanthes quinquangulata* A.Gray	1	*P. Phonsena, W. de Wilde & B. Duyfjes* 4416 (L)	Thailand, Phetchaburi	HE661374	HE661534	HE661452	-	-	-
*Trichosanthes quinquangulata* A.Gray	2	*N. Koonthudthod* et al. 326 (L)	Thailand, Phetchaburi	HE661375	HE661535	HE661453	-	-	-
*Trichosanthes quinquefolia* C.Y.Wu		*K. Nanthavong* et al. BT 705 (L)	Laos, Khammouan	HE661376	HE661536	HE661454	-	-	-
*Trichosanthes reticulinervis* C.Y.Wu ex S.K.Chen		*X.F. Deng* 131 (IBSC)	China, Guangdong	HE661377	DQ536605	DQ536744	DQ535857	DQ536876	DQ536876
*Trichosanthes rosthornii* Harms	1	*Jingliang Chuan* 5654 (KUN)	China	HE661378	HE661537	HE661455	-	-	-
*Trichosanthes rosthornii* Harms	2	*A. Henry* 1626 (LE)	China, Hubei	HE661379	HE661538	-	-	-	-
*Trichosanthes schlechteri* Harms		*W. Takeuchi & D. Ama* 15663 (LAE)	Papua New Guinea	HE661380	EU155619	EU155610	EU155605	EU155628	EU155638
*Trichosanthes sepilokensis* Rugayah		*J. Postar* et al. SAN 151201 (L)	Malaysia, Borneo, Sabah	HE661381	HE661539	-	-	-	-
*Trichosanthes smilacifolia* C.Y.Wu		*Qiwu Wang* 85620 (KUN)	China	HE661382	HE661540	-	-	-	-
*Trichosanthes subvelutina* F.Muell. ex Cogn.	1	*I. Telford* 9778 (CANB)	Australia, Queensland	HE661383	HE661541	HE661456	-	-	-
*Trichosanthes subvelutina* F.Muell. ex Cogn.	2	*F. Davies* 1541 (CANB)	Australia, Queensland	HE661384	HE661542	HE661457	-	-	-
*Trichosanthes subvelutina* F.Muell. ex Cogn.	3	*N. Nicholson* 3110 (BRI)	Australia, New South Wales	HE661385	HE661543	HE661458	-	-	-
*Trichosanthes tricuspidata* Lour spp. *javanica* Pruesapan & Duyfjes		*P. Phonsena, W. de Wilde & B. Duyfjes* 4414 (L)	Thailand, Phetchaburi	-	HE661592	HE661591	-	-	-
*Trichosanthes tricuspidata* Lour. ssp. *tricuspidata*		*P. Phonsena, W. de Wilde & B. Duyfjes* 4007 (L)	Thailand, Nakhon Sawan	HE661386	HE661544	HE661459	-	-	-
*Trichosanthes truncata* C.B.Clarke	1	*P. Phonsena, W. de Wilde & B. Duyfjes* 3917 (L)	Thailand, Chiang Mai	HE661387	HE661545	HE661460	-	-	-
*Trichosanthes truncata* C.B.Clarke	2	*P. Phonsena, W. de Wilde & B. Duyfjes* 4490 (L)	Thailand, Chiang Mai	HE661388	HE661546	HE661461	-	-	-
*Trichosanthes truncata* C.B.Clarke	3	*P. Phonsena, W. de Wilde & B. Duyfjes* 6329 (L)	Thailand, Chiang Mai	HE661389	HE661547	HE661462	-	-	-
*Trichosanthes villosa* Blume	1	*P. Phonsena, W. de Wilde & B. Duyfjes* 4669 (L)	Thailand, Chiang Mai	-	EU037006	EU037007	EU037005	EU037009	EU037008
*Trichosanthes villosa* Blume	2	*P. Phonsena, W. de Wilde & B. Duyfjes* 6331 (L)	Thailand, Chiang Mai	HE661390	: HE661548	HE661463	-	-	-
*Trichosanthes villosa* Blume	3	*P. Phonsena, W. de Wilde & B. Duyfjes* 4449 (L)	Thailand, Chiang Mai	HE661391	HE661549	HE661464	-	-	-
*Trichosanthes villosa* Blume	4	*P. Phonsena, W. de Wilde & B. Duyfjes* 4000 (L)	Thailand, Phetchaburi	HE661392	HE661550	-	-	-	-
*Trichosanthes villosa* Blume	5	*K. Pruesapan* et al. 60 (L)	Thailand, Kanchanaburi	HE661393	HE661551	HE661465	-	-	-
*Trichosanthes fissibracteata* C.Y.Wu ex C.Y.Cheng & Yueh		*Shaowen Yu* 974 (KUN)	China, Yunnan	HE661394	HE661552	HE661466	-	-	-
*Trichosanthes wallichiana* (Ser.) Wight		*A. Henry* 9432 (LE)	China, Yunnan	HE661395	HE661553	-	-	-	-
*Trichosanthes wawrae* Cogn.		*B. Gravendeel* et al. 631 (L)	Indonesia, Java	HE661396	HE661554	HE661467	-	-	-

Total DNA was extracted using the Carlson/Yoon DNA isolation procedure [45] and a Mini-Beadbeater (BioSpec Products) to pulverize the plant material. Extracts were purified using the GE Illustra GFX™ PCR DNA and Gel Band Purification Kit following the standard protocol.

Polymerase chain reaction (PCR) amplification of purified total DNA was performed in 200 μl reaction tubes with a total volume of 50 μl. Each tube contained a mixture of 5 μl reaction buffer (ABgene, 10x), 3 μl MgCl2 (25 mM), 1 μl dNTP’s (10 μM), 0.25 μl Taq-polymerase (ABgene; 5U/μl), 0.25 μl BSA (Roche Diagnostics), 12.5 μl of each primer (2 mM), 14.5 μl Milli-Q water and 1 μl template DNA. The ITS region was amplified using the primer pair ITS-P17 and ITS-26 S-82R [46] with the following PCR protocol 97°C 5 min., (97°C 30 s., 55°C 1 min., 72°C 1 min.) x 35, 72°C 10 min., 4°C ∞; *matK* with primers *matK*-2.1a [47] and *matK*-1440R [48], 95° 5 min., (95° 30 s., 50° 1 min., 72° 1 min.) x 35, 72° 10 min., 4° ∞; and *rpl20rps12* using the primers *rpl20* and *rps12*[49], 95° 5 min., (95° 30 s., 53° 1 min., 72° 1 min.) x 35, 72° 10 min., 4° ∞. Sequencing was performed by Macrogen Inc. (Seoul, South Korea) on an ABI3730XL automated sequencer (Applied Biosystems). The same primers as used in the PCR were used for the sequencing reactions.

### Sequence alignment

Sequence trace files were compiled into contigs with the program Gap4 and edited using Pregap4 [50], both part of the Staden package [51]. Sequences were aligned manually in Se-Al [52]. The final matrix included *rpl20-rps12* (100% of taxa), ITS (96%), *matK* (84%), *trnL-F* spacer (31%), *trnL* intron (28%), and *rbcL* (20%). The three latter regions increased statistical support values at early-branching clades. Sequences were concatenated, and gap-coded using the Simmons and Ochoterena simple method [53] implemented in SeqState [54].

### Phylogenetic analyses

Selection of best-fit models of nucleotide substitution for the nuclear and plastid data partitions relied on the Akaike Information Criterion (AIC and AICc) as implemented in JModelTest version 0.1.1 [55,56]. Likelihood calculations were carried out for 88 substitution models on an ML-optimized tree. The best-fitting model for the combined data was the general time-reversible (GTR) model, with a proportion of invariable sites (I) and rate variation among sites (G) with four rate categories. Maximum likelihood tree searches and bootstrapping of the combined data (using 1000 replicates) relied on RAxML version 7.2.6 [57] on the CIPRES cluster [58].

Bayesian tree searching used MrBayes [59] on the CIPRES cluster [58]. The combined data were analyzed using three partitions (nuclear, plastid, gap data), allowing partition models to vary by unlinking gamma shapes, transition matrices, and proportions of invariable sites. Markov chain Monte Carlo (MCMC) runs started from independent random trees, were repeated twice, and extended for 10 million generations, with trees sampled every 1000th generation. We used the default priors in MrBayes, namely a flat Dirichlet prior for the relative nucleotide frequencies and rate parameters, a discrete uniform prior for topologies, and an exponential distribution (mean 1.0) for the gamma-shape parameter and branch lengths. Convergence was assessed by checking that the standard deviations of split frequencies were <0.01; that the log probabilities of the data given the parameter values fluctuated within narrow limits; that the convergence diagnostic (the potential scale reduction factor given by MrBayes) approached one; and by examining the plot provided by MrBayes of the generation number versus the log probability of the data. Trees saved prior to convergence were discarded as burn-in (10 000 trees) and a consensus tree was constructed from the remaining trees.

The data matrix and trees have been deposited in TreeBASE (http://www.treebase.org; study number 12339).

### Divergence time estimation

Divergence times were estimated using the Bayesian relaxed clock approach implemented in BEAST version 1.6.2 [60]. Searches used a Yule tree prior, the GTR + G substitution model, and 50 million MCMC generations, sampling every 1000th generation. Six monophyletic groups were defined based on the results of our phylogenetic analyses and previously published phylogenies [18,20,44]. Tracer version 1.5 [61] was used to check that effective sampling sizes had all reached >200, suggesting convergence of the chains. TreeAnnotator, part of the BEAST package, was then used to create a maximum clade credibility tree, with the mean divergence ages shown for all nodes with >95% highest posterior density.

Calibration relied on Cucurbitaceae fossils assigned to particular nodes (labeled A--C in Figure 3), using a gamma prior distribution with the fossil age as the offset and shape and scale parameter chosen to add a 95% CI of c. 10 Ma older than the fossil. (A) The root node, that is, the most recent common ancestor of *Momordica* and *Trichosanthes*, was constrained to 55.8 Ma with a shape parameter of 1.0 and a scale of 1.0, based on seeds from the Paleocene/Eocene Felpham flora representing the oldest Cucurbitaceae and dated to c. 55.8 Ma [62]. (B) The crown node of the *Trichosanthes*/*Gymnopetalum* clade was constrained to 34 Ma with a shape parameter of 1.0 and a scale of 3.4, based on *Trichosanthes* seeds from the Upper Eocene of Bulgaria [25] dated to c. 34 Ma and seeds from the Oligocene of West Siberia [26] dated to c. 23.8 Ma [27]. (C) The divergence of *Marah* and *Echinocystis* was set to 16 Ma with a shape parameter of 1.0 and a scale of 3.35, based on leaves and a fruit representing *Marah* from the Miocene of Stewart Valley, Nevada (M. Guilliams and D. M. Erwin, University of California, Berkeley, in preparation; the fruit comes from the Fingerrock Wash site, dated to c. 16 Ma, the leaf from the Savage Canyon Formation, dated to c. 14.5 Ma). Absolute ages were taken from the geologic time scale of Walker and Geissman [63]. We also tested lognormal and exponential prior distributions, which gave very similar age estimates (results not shown).

### Biogeographical analysis

Biogeographic reconstruction relied on statistical dispersal-vicariance analysis using S-DIVA version 2.0 [64] as implemented in RASP, which carries out parsimony inference on the chain of trees obtained from an MCMC search [65,66], in our case the 8000 post burn-in Bayesian trees resulting from the BEAST dating analysis. S-DIVA averages the frequencies of an ancestral range at a node in ancestral reconstructions over all trees, with alternative ancestral ranges at a node weighted by the frequency of the node [64]. Range information for all species was compiled from taxonomic treatments [9,11,13-16], and the coded distribution areas were: A) Australia and New Guinea, B) Wallacea, C) Insular Sunda Malesia, D) Mainland Southeast Asia, E) India and adjacent countries, F) Africa, Europe and the New World.

## Authors’ contributions

HB conceived the study, carried out the molecular genetic analyses, and drafted the manuscript. HS participated in the design of the study and data analysis, and also contributed field observations. SR and MT participated in the design and coordination of the study, and SR also helped with clock calibration and writing. All authors read and approved the final manuscript.
